# Erkenntnisse aus 31 Stunden Stromausfall in Berlin Köpenick – medizinische Schwerpunkte und Herausforderungen

**DOI:** 10.1007/s00101-021-00930-x

**Published:** 2021-02-23

**Authors:** Florian Breuer, Paul Brettschneider, Per Kleist, Stefan Poloczek, Christopher Pommerenke, Janosch Dahmen

**Affiliations:** 1 Berliner Feuerwehr, Voltairestr. 2, 10179 Berlin, Deutschland; 2 Ärztliche Leitung, Rettungsdienst im Land Berlin, Berlin, Deutschland; 3 Fakultät für Gesundheit, Universität Witten Herdecke, Witten, Deutschland

**Keywords:** Vulnerabilität, Großschadensereignis, Vorplanung, Vorsorge, Selbsthilfe, Vulnerability, Major disaster, Preliminary planning, Prevention, Self-help

## Abstract

Am 19.02.2019 kam es nach Durchtrennung eines 110-kV-Kabels zu einem großflächigen Stromausfall im Berliner Bezirk Treptow-Köpenick. Nachfolgend waren ca. 30.000 Haushalte ohne Strom; betroffen waren ca. 70.000 Menschen. Der Stromausfall dauerte mehr als 24 h an und stellte alle Beteiligten vor eine Vielzahl von Herausforderungen. Es wurde der Führungsstab (operativ-taktisch) einberufen, in dem auch medizinische Schwerpunkte fortlaufend identifiziert und reevaluiert werden mussten. Hierbei handelte es sich mitunter um die Identifikation von besonders gefährdeten Patienten wie beispielsweise heimbeatmeten Patienten oder Patienten mit Kunstherz. Weiterhin mussten einzelne Pflegeheime evakuiert werden. Im Verlauf war es notwendig, aufgrund des Ausfalls der Stromversorgung im Schadensgebiet, eine Intensivstation bzw. „Intermediate-care“-Station mit 23 Patienten zu evakuieren. Krankenhäuser müssen sich im Rahmen von Vorplanungen auf derartige Szenarien einstellen. Weiterhin müssen seitens der zuständigen Behörden Vorplanungen erfolgen, die die besonderen Bedürfnisse von vulnerablen Gruppen beinhalten.

## Abstract (en)

On 19 February 2019 the severance of a 110kW cable caused an extensive electrical power cut in the Treptow-Köpenick district of Berlin. Subsequently, ca. 30,000 households were without electricity and ca. 70,000 people were affected. The power cut lasted more than 24h and all those involved were faced with a multitude of challenges. An operational command post was set up in which medical problems had to be continuously identified and re-evaluated. These included the identification of patients particularly at risk, such as home-ventilated patients and patients with artificial hearts. Furthermore, individual nursing homes had to be evacuated. During the procedure it was necessary to evacuate an intensive care ward or intermediate care ward with 23 patients due to the loss of power supply in the affected area. Hospitals must be prepared for such scenarios within the framework of preliminary planning. Furthermore, preliminary planning containing the special needs of vulnerable groups must be carried out on the part of the responsible authorities.

## Hintergrund

Zwar gilt das deutsche Stromnetz insgesamt als sehr stabil und zuverlässig, jedoch sind Blackout-Szenarien auch in Deutschland möglich. Auch wenn die meisten Stromausfälle lokal begrenzt sind und binnen weniger Stunden behoben werden können, besteht die besondere Problematik dennoch darin, dass derartige Szenarien in Abhängigkeit von der Ursache großräumig und lang andauernd sein können und somit insbesondere vulnerable Gruppen gefährden.

Ursächlich sind insbesondere Extremwetterereignisse sowie instabile Netze, beispielsweise aufgrund von Überlastung. Auch Hackerangriffe stellen zunehmend eine potenzielle Bedrohung dar. So kam es beispielsweise im September 2020 zu einem Hackerangriff auf die Uniklinik Düsseldorf, wodurch es zu einem Komplettausfall der Informationstechnik (IT) kam, was dazu führte, dass die Notaufnahme schließen musste, Operationstermine verschoben wurden und Behandlungstermine abgesagt werden mussten. Das Krankenhaus musste in der Folge für 13 Tage von der Versorgung abgemeldet werden [13]. Bereits 2016 war über einen abgewehrten Hackerangriff auf ein Krankenhaus in Neuss berichtet worden, der mit massiven Einschränkungen des Betriebes einherging. Regelmäßig kommt es darüber hinaus nahezu täglich zu Beschädigungen von Leitungen durch Tiefbauarbeiten. Diese können jedoch durch Schaltmaßnahmen der Netzbetreiber in der Regel auf wenige Minuten begrenzt werden. In den USA werden immer wieder großflächige und teilweise lang andauernde Stromausfälle infolge von Unwetterereignissen wie Hurrikanen beschrieben [9, 19, 31].

Nach Angaben der Versorgungs- und Zuverlässigkeitsstatistik des Forum Netztechnik/Netzbetrieb betrug die durchschnittliche Stromunterbrechungsdauer in Deutschland im Jahr 2018 13,3 min/Kunde (2019: 12,0 min); unter Berücksichtigung von Fällen höherer Gewalt lag diese bei 17,1 min. Hiernach verfügt Deutschland auch im Vergleich zu anderen Ländern sowohl innerhalb als auch außerhalb Europas über eine hohe Stabilität der Stromnetze [17].

Dennoch muss nach der Einschätzung des Bundesamtes für Bevölkerungsschutz und Katastrophenhilfe (BBK) auch in Deutschland mit Stromausfällen über längere Zeiträume gerechnet werden. Dies zeigte sich eindrücklich im Jahr 2005 im Münsterland, als, bedingt durch den Wintereinbruch, teilweise bis zu 20.000 Haushalte 5 Tage oder mehr ohne Strom auskommen mussten [6].

Hierbei ist zu berücksichtigen, dass auch die Funktionalität der Informations- und Kommunikationsnetze beeinträchtigt sein kann. Zwar halten die Telekommunikationsanbieter Reservekapazitäten vor, dennoch sind auch diese im Rahmen der Abhängigkeit von Netzersatzanlagen oder Notstromaggregaten nur begrenzt verfügbar. Weiterhin ist damit zu rechnen, dass es aufgrund eines vermehrten Informations- und Kommunikationsbedarfs aufgrund von Überlastung zu Ausfällen kommt. Es ist zu bedenken, dass bei der Festnetztelefonie die Basisstationen für die Schnurlostelefone auf eine funktionierende Stromversorgung angewiesen sind und es somit hier schnell zu Ausfällen kommt. Ortsvermittlungsstellen und Fernvermittlungsstellen sind ebenfalls von einer kontinuierlichen Stromversorgung abhängig, wobei hier Reservekapazitäten von 15 min bis zu 8 h (Ortsvermittlungsstellen) sowie von 8 h bis zu 3 bis 4 Tagen (Fernvermittlungsstellen) möglich sind. Beim Mobilfunk ist eine Einwahl in eine Basisstation notwendig, die nach 15 min bis 8 h ausfällt. Vermittlungsstellen halten Reservekapazitäten von 8–48 h vor [27]. Auch wenn Notrufe zwar im Telefonvermittlungssystem privilegiert behandelt werden, so ist es dennoch zwingend Voraussetzung, dass das Endgerät eine Verbindung zum Telefonnetz aufbauen kann.

Auch der bei den Behörden und Organisationen mit Sicherheitsaufgaben (BOS) genutzte Digitalfunk ist auf ausreichende Akkureserven und auf eine funktionierende Notstromversorgung angewiesen. Eine Notstromversorgung kann den Betrieb für eine Dauer bis zu 72 h sicherstellen.

Im Ratgeber für Notfallvorsorge und richtiges Handeln in Notsituationen des BBK wird die Bevölkerung aufgerufen, individuelle Vorkehrungen für einen Stromausfall zu treffen; weiterhin werden Verhaltenstipps insbesondere bei ausgefallener Heizung oder fehlendem Licht gegeben [5]. Es muss jedoch klar sein, dass eine derartige Selbsthilfe nur von einem bestimmten Anteil der Bevölkerung geleistet werden kann und insbesondere vulnerable Gruppen sowohl im Rahmen der Vorplanungen als auch bei einem derartigen Ereignis einer erhöhten Aufmerksamkeit bedürfen.

## Stromausfall in Berlin-Köpenick – Szenario

**Figure 1:**
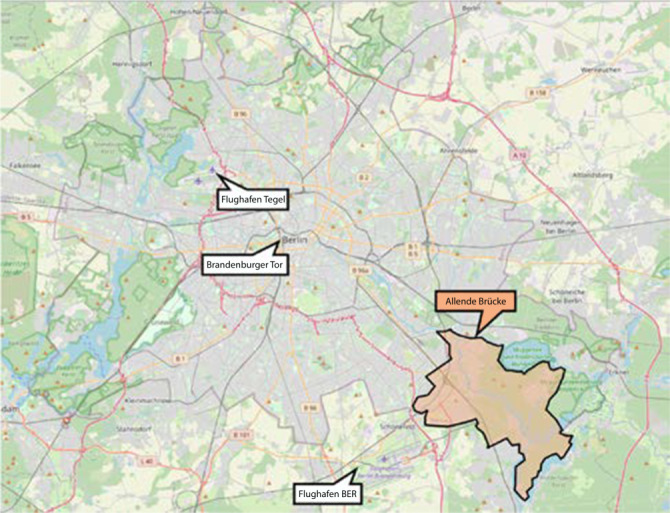

Im Rahmen von Bauarbeiten auf der Salvador-Allende-Brücke in Berlin-Köpenick wurde am Dienstag, den 19.02.2019 um 14:10 Uhr ein 110-kV-Kabel des Mittelspannungsnetzes durchtrennt. Betroffen war in der Folge der hinter der Brücke liegende Netzbereich des Bezirkes Treptow-Köpenick (Abb. 1). Dieser umfasst eine Fläche von 75 km^2^, ca. 30.000 Haushalte, wobei wiederum ca. 70.000 Menschen betroffen waren. Neben einer Vielzahl von Gewerbebetrieben sind in dem Gebiet 2 Krankenhäuser der Grund- und Regelversorgung, aber auch eine Vielzahl von Pflegeeinrichtungen angesiedelt. Die Temperatur schwankte an dem Tag zwischen 2 und 10 °C.

Erste Informationen erhielt der Lagedienst der Feuerwehr durch den örtlichen Netzbetreiber telefonisch um 14:20 Uhr. Bereits um 14:21 Uhr (11 min nach Ereignisbeginn) kam es in einer Intensivpflegeeinrichtung zum ersten ereignisassoziierten Einsatz.

Der Einsatz lässt sich retrospektiv in 4 Phasen beschreiben (Tab. 1; [1]).

**Table 1:** 

Phase 1 (erste 2 h)	Vermehrt Notrufe aus dem Schadensgebiet Hilfeersuchen einer ambulanten Intensivpflegeeinrichtung
Phase 2 (Stunden 2–8)	Sicherstellung des Notrufs im Schadensgebiet Sicherstellung der Kommunikations- und Alarmierungswege Sicherstellung der medizinischen Versorgung im Schadensgebiet und Aufrechterhaltung der Betriebsfähigkeit beider Krankenhäuser Information der Bevölkerung und Hilfe zur Selbsthilfe
Phase 3 (Stunden 8–24)	Einrichtung von Anlaufstellen für Betroffene durch das Bezirksamt Kostenloser Transport durch die Berliner Verkehrsbetriebe im bzw. aus dem Schadensgebiet heraus Identifikation von vulnerablen Patienten Aufsuchen von Pflegeheimen und Privatadressen durch Erkunder und Unterstützung vor Ort Verlegung von 23 Intensivpatienten (18 beatmet) aus dem betroffenen Krankenhaus in umliegende Krankenhäuser
Phase 4 (mehr als 24 h)	Aufsuchen von vulnerablen Patientengruppen Sicherstellung der Heizungsversorgung in verschiedenen Einrichtungen (z. B. Pflegeheime) Einrichtung einer Notunterkunft für 250 Personen durch das Bezirksamt Herausgabe von Verpflegung an 6 Standorten durch das Bezirksamt, unterstützt durch Hilfsorganisationen Sicherung der Abwasserentsorgung Wiederherstellung der Stromversorgung

In der *Phase 1 (erste 2* *h)* wurde ein genaues Lagebild beim Netzbetreiber angefordert, der dann mitteilte, dass die Reparaturarbeiten voraussichtlich bis zu 24 h andauern würden. Somit wurde um 16 Uhr (1 h 50 min nach Ereignisbeginn) durch den Gesamteinsatzleiter nach Rücksprache mit der Behördenleitung ein operativ-taktischer Stab (Stab Feuerwehr) einberufen.

In der *Phase 2 (Stunden 2–8)* übernahm zunächst um 17:00 Uhr (2 h 50 min nach Ereignisbeginn) der Stab Feuerwehr den Gesamteinsatz, sodass in Abstimmung mit dem A‑Dienst (höchster Einsatzleiter der Feuerwehr) strategische Ziele definiert wurden. Zuvor war aufgrund des Einsatzstichwortes „Stabsalarm“ zunächst übergangsweise eine Stabsbesetzung aus Funktionen, die sich bereits im Einsatzdienst befinden, erfolgt.

**Figure 2:**
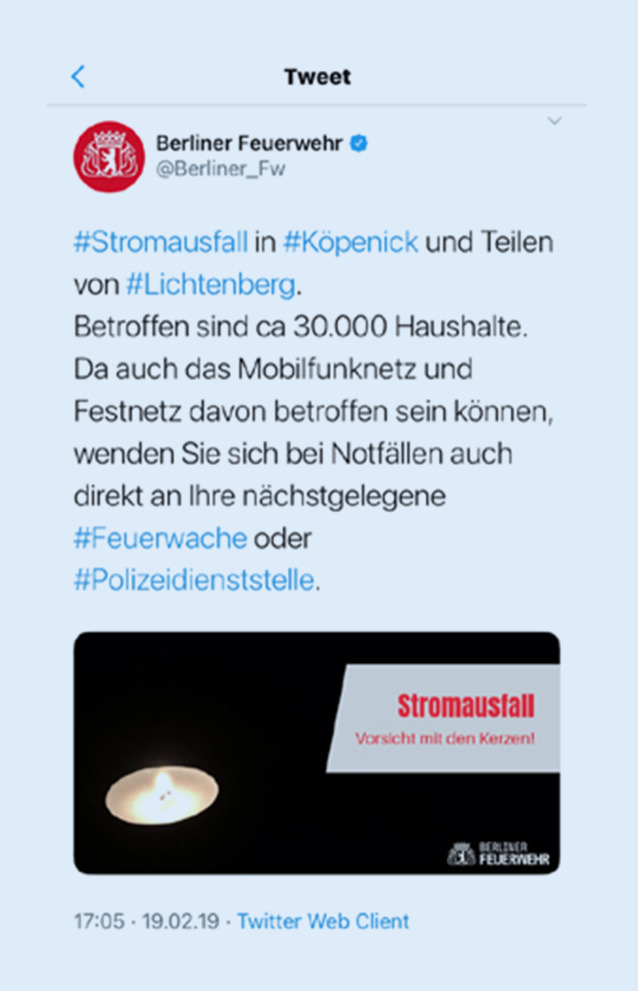

Es wurden dann zunächst Kräfte der Freiwilligen Feuerwehren (6 Freiwillige Feuerwehren), des Technischen Hilfswerks (THW) (2 Fachgruppen Elektroversorgung, eine Fachgruppe Infrastruktur, 2 Bergungsgruppen, eine Fachgruppe Wasserschaden/Pumpen) und der Hilfsorganisationen (4 Patiententransportzüge für jeweils 10 Patienten, 2 Betreuungsplätze für jeweils 500 Personen) zur Bereitstellung in ihren Unterkünften alarmiert. Weiterhin erfolgte unter Zuhilfenahme der sozialen Medien die Information der Bevölkerung. Die Kommunikation erfolgte über Nachrichtendienste wie Twitter oder Facebook. Hierbei handelte es sich insbesondere um Handlungsempfehlungen, aber auch um den Hinweis, dass Hilfeersuchen an die nächstgelegene Feuer- oder Polizeiwache gerichtet werden sollen (Abb. 2). Zusätzlich wurden auch Fahrzeuge zur Erkundung entsendet oder als mobile Wachen als Anlaufstelle für die Bevölkerung zentral aufgestellt (insgesamt 9 mobile Anlaufstellen). Darüber hinaus dienten Feuerwehrhäuser im Schadensgebiet als Anlaufpunkte für die Bevölkerung.

Es erfolgte dann die Identifizierung von vulnerablen medizinischen Einrichtungen im Schadensgebiet: Hierbei konnten 2 Krankenhäuser der Grund- und Regelversorgung (525 und 441 Betten), 5 Pflegeheime, ein Hospiz sowie eine Dialyseeinrichtung identifiziert werden. Zu allen Einrichtungen wurden Erkunder entsendet.

Es gab des Weiteren umfangreiche Bemühungen, aufgrund der Abhängigkeit vom Stromnetz besonders gefährdete Patienten im Schadensgebiet zu identifizieren. Hierzu gehören u. a. Patienten mit Kunstherz, Heimbeatmungsgerät oder Heimdialyse, aber auch andere organisierte Wohnformen (Alten-Wohnen, private Intensivpflege), die nicht unter das Wohn- und Teilhabegesetz fielen und deshalb nicht zentral über die landesweite Heimaufsicht identifiziert werden konnten. Die Identifikation von Patienten mit Kunstherz gelang mithilfe des Deutschen Herzzentrums Berlin (DHZB), da dieses über eine medizinische und technische Hotline für dringende Fragen von entsprechend angebundenen Patienten oder behandelnden Ärzten verfügt. Darüber hinausgehende Informationen zu weiteren hilfsbedürftigen Patienten waren nicht ohne Weiteres herauszubekommen. Nahezu vollumfänglich war eine telefonische Kontaktaufnahme (Festnetz, Mobilfunk, Hausnotruf) mit Menschen im Schadensgebiet nicht möglich. Hierdurch erfolgte einerseits eine Vielzahl von Anrufen besorgter Angehöriger aus anderen Bezirken in der Leitstelle, weiterhin war konsekutiv auch eine Vielzahl von Erkundungen notwendig.

Gegen 7 h 10 min nach Ereignisbeginn erfolgte sodann die Meldung eines der beiden Krankenhäuser, dass die Stromversorgung instabil ist. Daraufhin wurde unverzüglich eine Fachgruppe des THW dorthin entsendet, um eine Notstromversorgung aufzubauen. Um 21:45 Uhr (7 h 35 min nach Ereignisbeginn) erfolgte schließlich die Meldung an den Stab, dass die Notstromversorgung des Krankenhauses ganz zusammengebrochen war. Eine alternative Einspeisung war zu diesem Zeitpunkt noch nicht absehbar.

**Figure 3:**
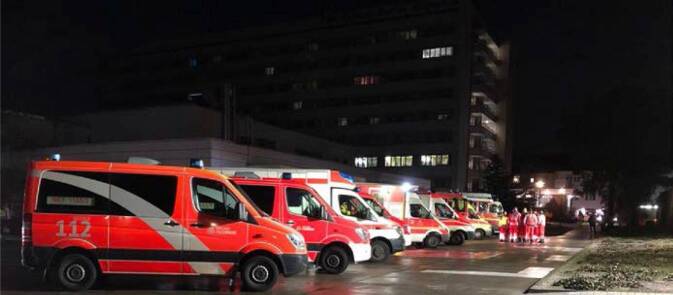

In der *Phase 3 (Stunden 8–24) *wurde im operativ-taktischen Stab unter Einbeziehung der Krankenhauseinsatzleitung sowie der Senatsverwaltung für Gesundheit, Pflege und Gleichstellung entschieden, dass die 23 Intensivpatienten (18 beatmet) aus dem betroffenen Krankenhaus in umliegende Krankenhäuser verlegt werden müssen. Dies erfolgte dann ab 22:21 Uhr (8 h 11 min nach Ereignisbeginn). Die Transportorganisation vor Ort erfolgte durch einen leitenden Notarzt (LNA), sodass nach 3 h alle Intensivpatienten verlegt waren (Tab. 2). Hierzu war die zusätzliche Indienststellung von Fahrzeugen des Spitzenbedarfs (= zusätzlicher kurzfristiger Bedarf an Rettungsmitteln bei besonderen Schadenslagen) notwendig, weiterhin erfolgte die zusätzliche Gestellung von Ärzten durch ärztliche Einsatztrupps, die seitens der Krankenhäuser zur Unterstützung bei derartigen Schadensereignissen vorgehalten werden müssen (Abb. 3).

**Table 2:** 

Lfd. Nr.	Uhrzeit	Station	Geschlecht/Alter	Anamnese	Transport durch
1	22:45–04:00 Uhr	Kreißsaal	w	Beginnende Geburt zur Sectio	RTW
2	22:45–04:00 Uhr	ITS	w/66 J	ARDS (Pneumonie), intubiert	ITW
3	22:45–04:00 Uhr	ITS	w/57 J	ARDS (Pneumonie), intubiert	ITW
4	22:45–04:00 Uhr	ITS	w/68 J	Peritonitis, intubiert	ITW
5	22:45–04:00 Uhr	ITS	m/83 J	Exazerbierte COPD, intubiert	ITW
6	22:45–04:00 Uhr	ITS	w/68 J	Sepsis (nekrotisierende. Pankreatitis), intubiert	NEF+RTW
7	22:45–04:00 Uhr	ITS	w/81 J	Akutes Nierenversagen, intubiert	NEF+RTW
8	22:45–04:00 Uhr	ITS	w/77J	Exazerbierte COPD, intubiert	NEF+RTW
9	22:45–04:00 Uhr	ITS	w/78J	Zustand nach Oberschenkelhalsfraktur, Dysphagie, tracheotomiert	NEF+RTW
10	22:45–04:00 Uhr	ITS	m/80 J	Kardiale Dekompensation, intubiert	NEF+RTW
11	22:45–04:00 Uhr	ITS	m/64 J	Nekrotisierende Pankreatitis, intubiert	NEF+RTW
12	22:45–04:00 Uhr	ITS	w/85 J	Zustand nach Reanimation, intubiert	NEF+RTW
13	22:45–04:00 Uhr	ITS	w/74 J	Peritonitis, intubiert	NEF+RTW
14	22:45–04:00 Uhr	ITS	m/74 J	Autoimmunvaskulitis, High-Flow-Ventilation	NEF+RTW
15	22:45–04:00 Uhr	ITS	w/33 J	Pneumonie, intubiert	NEF+RTW
16	22:45–04:00 Uhr	ITS	w/82 J	Dekompensierte Herzinsuffizienz, intubiert	Arzt+RTW
17	22:45–04:00 Uhr	ITS	w/78 J	Akutes Koronarsyndrom, heimbeatmet	ITW
18	22:45–04:00 Uhr	IMC	w/73 J	Äthyltoxisches Leberversagen, tracheotomiert	KTW
19	22:45–04:00 Uhr	IMC	m/60 J	Oropharynx-CA (palliativ)	RTW
20	22:45–04:00 Uhr	IMC	w/77 J	Gastrointestinale Blutung unter oralen Antikoagulanzien	RTW
21	22:45–04:00 Uhr	IMC	w/68 J	Pneumonie	RTW
22	22:45–04:00 Uhr	IMC	m/76 J	Kardiale Dekompensation/COPD	RTW
23	22:45–04:00 Uhr	IMC	m	Tachyarrhythmia absoluta/Vorhofflimmern	RTW

In der *Phase 4 (mehr als 24* *h) *war seitens des Netzbetreibers angekündigt, dass an diesem Tag die Stromversorgung wiederhergestellt werden soll. Schlussendlich war der Netzstrom allerdings erst am Folgetag um 21:30 Uhr (31 h 20 min nach Ereignisbeginn) wieder vollumfänglich verfügbar. Weiterhin erfolgten die Vorplanung der Rücktransporte der Patienten in das betroffene Krankenhaus.

## Krankenhäuser als kritische Infrastruktur beim Stromausfall

Krankenhäuser sind im Kritische-Infrastruktur(KRITIS)-Sektor Gesundheit dem Bereich medizinische Versorgung zugeordnet und sind somit neben Arzneimitteln und Impfstoffen sowie Laboren besonders schützenswert. Hierbei gibt es eine Vielzahl von Schnittstellen und Abhängigkeiten, insbesondere zum Rettungsdienst, der dem KRITIS-Sektor „Staat und Verwaltung“ zugehörig ist, aber auch zu dem KRITIS-Sektor „Transport und Verkehr“.

Es ist davon auszugehen, dass Krankenhäuser unter Zuhilfenahme von Notstromaggregaten bei einem Stromausfall bis zu 24 h autark weiterarbeiten können, danach ist die Funktionsfähigkeit aufgrund der Erschöpfung der eigenen Notstromversorgung (eingeschränkte Treibstoffversorgung) erheblich beeinträchtigt. Hierbei erfolgen die konkreten Festlegungen durch landesrechtliche Regelungen, wobei weitergehende Vorsorgemaßnahmen wie die Vorhaltung von größeren Tanks auf freiwilliger Basis vorgenommen werden. Ausfälle weiterer KRITIS wie Transport und Versorgung verstärken die Problematik. Bei Andauern von mehr als einer Woche muss damit gerechnet werden, dass bei einem großflächigen Stromausfall trotz regionaler Unterstützung die Versorgungsstrukturen und die Infrastruktur zusammenbrechen.

Hierbei spielt die gestörte Produktion von Arzneimitteln genauso eine Rolle wie die Tatsache, dass Produkte wie Blutkonserven und Plasma bei nichtsachgerechter Lagerung verderblich sind. Weiterhin kommt es zu massiven Einschränkungen aufgrund der gestörten Kommunikationsinfrastruktur und der nicht mehr vorhandenen Transportdienstleistungen (Zusammenbruch des Straßenverkehrs, keine Verfügbarkeit von Kraftstoff an Tankstellen aufgrund fehlender Notstromversorgung). In dieser Phase ist es denkbar, dass einzelne, zentrale Krankenhäuser zu „zentralen Knotenpunkten der medizinischen Versorgung“ werden [12]. Diese müssen sich sodann darauf einstellen, eine Vielzahl von Patienten aus den umliegenden Krankenhäusern, aber auch aus Pflegeheimen und vergleichbaren Einrichtungen zu übernehmen.

Diejenigen Bereiche, die von der Notstromversorgung umfasst sind, zeigt (Infobox 1). Hierbei ist zu beachten, dass bei einem Stromausfall insbesondere das Rechenzentrum, die Küche, aber auch die Warmwasserversorgung nicht mehr funktionsfähig sind.

Die zu erwartenden Beeinträchtigungen in Krankenhäusern in Abhängigkeit von der vorangeschrittenen Zeit bei einem Stromausfall zeigt Tab. 3.

**Table 3:** 

Bis 2 h	Ausfall der Informations- und Kommunikationsstrukturen Organisatorische Beeinträchtigungen Apparative Diagnostik eingeschränkt
2–8 h	Ausfall der Bürotätigkeiten Arbeitsabläufe zunehmend behindert Patientendaten nicht mehr einsehbar bzw. bearbeitbar Überlastung der Telefonzentrale Ordnungsgemäße Lagerung von Arzneimitteln und Medizinprodukten gefährdet Küche nicht mehr funktionsfähig Trinkwasserknappheit
8–24 h	Deutliche Beeinträchtigung der medizinischen Versorgung Notwendigkeit der Entlassung von Leichtverletzten/Genesenen Notwendigkeit der Einrichtung von Behelfskrankenhäusern Patientendokumentation nur noch manuell Zahl der Selbsteinweiser steigt Gestörte Speiseversorgung
Mehrere Tage	Engpässe bei der Versorgung mit Blutprodukten, Insulin, Spezialnahrung Einzelne Medikamente knapp Hygienische Probleme aufgrund von beeinträchtigter Entsorgung

## Vulnerable Gruppen

In einer Umfrage im Nachgang zum Stromausfall im Münsterland wurden mit einem Zeitverzug von einem Jahr 591 Haushalte im Schadensgebiet bezüglich der Selbsthilfefähigkeit und des Bevorratungsverhaltens befragt. Inzwischen wird seitens des BBK empfohlen, einen 10-tägigen Grundvorrat von Getränken und Lebensmitteln sowie insbesondere eine Hausapotheke, Hygieneartikel sowie Vorkehrungen für einen Energieausfall vorzuhalten. Im Ergebnis zeigte sich u. a., dass trotz des erlebten Stromausfalls nur 27,7 % der befragten anders bevorraten als zuvor. Somit wurde von 72 % der Betroffenen keine Notwendigkeit einer eigenen Bevorratung gesehen, was zeigt, dass sich ein Großteil der Bevölkerung komplett auf die öffentliche Daseinsvorsorge und Gefahrenabwehr verlässt [24].

Im Auftrag des BBK wurde im Jahr 2012 eine umfassende Bevölkerungsbefragung durchgeführt, an der 2000 Haushalte teilgenommen haben. Es konnte gezeigt werden, dass sich 69 % aller Haushalte bislang noch nicht mit einem Stromausfallszenario befasst haben. 1,4 % aller Haushalte gaben darüber hinaus an, auf netzstrombetriebene, lebensnotwendige Geräte angewiesen zu sein (Sauerstoff‑/Beatmungsgerät 29,2 %, Sonstiges 22,2 %, Inhalationsgerät 9,7 %, Heimdialysegerät 7,9 %). In 32 % verfügen die Geräte über eine Notstromversorgung [29].

Eine Untersuchung in den USA ergab, bezogen auf die Anzahl derjenigen Menschen, die auf netzstrombetriebene Geräte angewiesen sind, eine Prävalenz von 218,2/100.000 Einwohner. Allerdings waren weniger als 1 % auf Heimbeatmungsgeräte angewiesen; die meisten waren abhängig von einer externen Sauerstoffzufuhr [25]. Auch in den USA konnte gezeigt werden, dass gerade vulnerable Gruppen unzureichend auf derartige Ereignisse vorbereitet sind und insbesondere damit einhergehende Risiken nicht hinreichend ernst genommen werden [15].

Die Betrachtung von vulnerablen Gruppen im Zusammenhang mit Stromausfällen zeigt, dass die Herausforderungen sehr komplex sind. Auch wenn eine Vielzahl von alten und pflegebedürftigen Menschen, die zu Hause oder in Heimen leben, besonders gefährdet ist, so handelt es sich dennoch um eine sehr heterogene Gruppe mit ganz unterschiedlichen Bedürfnissen [14]. Viele Menschen, insbesondere solche, die ohnehin schon im Alltag auf externe Hilfe angewiesen sind, benötigen bei einem Stromausfall umgehend personelle und materielle Unterstützung (Infobox 2). Hierbei stehen bei den einzelnen Gruppen jeweils ganz spezielle Bedürfnisse im Vordergrund. Hierbei ist auch zu berücksichtigen, dass viele ältere Menschen oder anderweitig immobilisierte oder mobilitätseingeschränkte Menschen nicht eigenständig mobil sind und somit auf Hilfsmittel sowie Aufzüge angewiesen sind. Auch ist die Vorratshaltung in Bezug auf Lebensmittel gerade in Großstädten sehr unterschiedlich und kann bei einem Vorratsmangel für diese vulnerable Gruppe schnell bedrohlich werden. Die Kommunikation ist auch sehr schnell eingeschränkt, da zum einen ein kompletter Ausfall vom Telefonnetz denkbar ist, zum anderen haben Akkus auch nur eine begrenzte Kapazität. Es ist zu berücksichtigen, dass viele Menschen auf ambulante Pflegedienste angewiesen sind. Dabei spielen sowohl die Körperhygiene als auch die Nahrungsaufnahme und die Medikamentengabe eine wichtige Rolle im Alltag. Somit müssen insbesondere diejenigen Menschen, die in der Häuslichkeit leben und von lebensnotwendigen Geräten abhängig sind, identifiziert werden (Infobox 3). Da es hierzu keine regionalen Melderegister gibt, gestaltet sich dies als besonders schwierig. In den USA konnte über die Meldedaten bei entsprechenden Versicherungsträgern eine Identifizierung von Personen, die ein Beatmungsgerät oder Sauerstoffgerät nutzen, erfolgen [11]. Aufgrund von datenschutzrechtlichen Vorgaben sind hier jedoch hierzulande Grenzen gesetzt.

Teilweise verfügen Pflegeheime oder vergleichbare Einrichtungen über eine Notstromversorgung für einige Stunden. Problematisch ist der Ausfall von Aufzügen und Türöffnern. Schnell kommt es zum Ausfall der Warmwasserversorgung und der Versorgung mit Lebensmitteln. Zeitnah werden insbesondere Verlegungen von denjenigen Bewohnern notwendig sein, die auf externe stromversorgte Geräte angewiesen sind.

## Diskussion

Bei dem Stromausfall in Berlin-Köpenick wurden die Berliner Feuerwehr und damit einhergehend die Berliner Notfallrettung vor eine Vielzahl von Herausforderungen gestellt. Neben 1346 Einsätzen am 19.02.2020 und 1313 Einsätzen am 20.02.2020 wurden insgesamt 112 Einsätze der Feuerwehr in Verbindung mit dem Stromausfall registriert.

Entscheidend war die frühzeitige Einberufung des Führungsstabs mit Etablierung einer Einsatzleitung, sodass von Beginn an auch medizinische Schwerpunkte identifiziert werden konnten. Dies wurde dadurch sichergestellt, dass der Oberarzt vom Dienst der Berliner Feuerwehr [4], neben den Sachgebietsleitern sowie Fachberatern und Verbindungsbeamten, dauerhaft im operativ-taktischen Stab der Feuerwehr anwesend war. Hierbei muss bei einem derartigen Szenario frühzeitig ein Schichtdienstplan erstellt werden, um dauerhaft über die Lage die entsprechende Ressource in die Entscheidungsfindungen einbinden zu können.

Die medizinischen Herausforderungen sind vielfältig und komplex. Ein großflächiger Stromausfall bedingt eine erhöhte Mortalität in der Bevölkerung durch die eingeschränkte Versorgung und damit einhergehend vermehrte Notlagen [2].

Infolge eines Stromausfalls muss damit gerechnet werden, dass es zu einer vermehrten Hospitalisierung, insbesondere von chronisch Kranken, kommt. Bei COPD-Erkrankten konnte nach einem Stromausfall sogar eine signifikant erhöhte Hospitalisierungsrate festgestellt werden [32]. Insbesondere im Nachgang zu einem derartigen Ereignis kann es zum vermehrten Aufsuchen von Notaufnahmen, aber auch zur erhöhten Auslastung bestimmter Funktionsbereiche, wie beispielsweise Dialysen kommen [21].

Aus lang andauernden Stromausfällen kann eine Vielzahl von gesundheitlichen Folgen resultieren, mitunter temperaturbedingte Erkrankungen, Magen-Darm-Erkrankungen, aber eben auch Erkrankungen des Herz-Kreislauf-Systems, der Atemwege oder Nierenerkrankungen mit der Notwendigkeit zur stationären Versorgung [8]. Somit ist damit zu rechnen, dass es auch schon in der Akutphase zu einem vermehrten Aufsuchen von Notaufnahmen kommt, da beispielsweise bei entsprechenden Patienten die Sauerstoffversorgung zu Hause nicht mehr sichergestellt ist, die entsprechenden Versorgungsdienste nicht erreicht werden können und somit Krankenhäuser zurate gezogen werden. Aufgrund des zunehmenden Informationsbedarfs muss in der Rettungsleitstelle mit einer massiven Steigerung des Anrufvolumens, insbesondere in den ersten Stunden nach Ereignisbeginn, gerechnet werden. So konnte im Nachgang eines Stromausfalls in Ohio gezeigt werden, dass in den ersten 10 h ein signifikant erhöhtes Anrufvolumen (durchschnittlich 250 % erhöht) insbesondere aufgrund von Anfragen bezüglich eines möglichen Ausfalls von elektrisch betriebenen medizinischen Geräten vorlag [28]. Auch mit einer erhöhten Auslastung des Rettungsdienstes muss gerechnet werden [20]. Hierbei sind aufgrund unsachgemäßer Nutzung von Notstromaggregaten, gasbetriebenen Heizungen und Grills auch wiederholt Kohlenmonoxidvergiftungen in Wohnungen beschrieben worden [22].

Problematisch ist, dass es keinerlei Melderegister gibt, welches Informationen zu besonderen Bedarfen von hilfsbedürftigen Personen enthält. Auch ist unklar, wo sich Pflegeheime mit speziellen Patientengruppen befinden (z. B. heimbeatmete Patienten). Hierbei ist auch zu berücksichtigen, dass sich diese Patientengruppe nicht nur in Pflegeheimen befindet, sondern auch in entsprechenden Pflegewohngemeinschaften oder aber in Privatwohnungen. Im Rahmen der Vorplanungen muss sich auf diese speziellen Bedürfnisse eingestellt werden, indem beispielsweise Pläne vorgehalten werden, die Informationen zu pflegebedürftigen Menschen und deren Bedarfen beinhalten.

In einer Umfrage aus dem Jahr 2019 gaben 68,4 % der zu Pflegenden und 80 % pflegender Angehöriger an, dass nach ihrer Ansicht bei einem Stromausfall Informationen durch die Feuerwehr zur Verfügung gestellt werden sollten. Bezogen auf die Sicherstellung der medizinischen Versorgung sahen pflegende Angehörige die Feuerwehr und den Rettungsdienst zu 59,1 % in der Pflicht. 45 % aller pflegenden Angehörigen gaben an, eine Notstromversorgung für die zu pflegende Person zu benötigen. Auch hier wurden in 65,6 % Feuerwehr und Rettungsdienst in der Pflicht gesehen. Von den zu Pflegenden gaben 13 % an, auf Strom betriebene medizinische oder andere Hilfsmittel angewiesen zu sein. Genannt wurden hierbei insbesondere Beatmungshilfen (36,8 %), Bett und Patientenlifter (22,4 %), Hausnotruf (9,1 %) sowie der elektrische Rollstuhl (7 %). 0,7 % gaben an, dass ein Stromausfall in Konsequenz zu einer lebensbedrohlichen Situation führen würde [30].

Krankenhäuser müssen als Bestandteil der KRITIS Notfallpläne für derartige Szenarien vorhalten. Hierbei sind die Herausforderungen und Besonderheiten auch innerhalb eines Krankenhauses ganz unterschiedlich. Somit sollten beispielsweise in der Anästhesie neben entsprechenden Checklisten Taschenlampen, geladene Akkus für die entsprechenden Medizingeräte sowie Back-up-Medizinprodukte- und -geräte vorgehalten werden, aber auch Experten kurzfristig erreichbar sein, die bei derartigen Szenarien technisch unterstützen können [16]. Auch die Verwendbarkeit von Medikamenten, Impfpräparaten oder Blutersatzprodukten kann nach Unterbrechung der Kühlkette eingeschränkt sein [23].

Auch sind Ausfälle von Notstromaggregaten in Krankenhäusern mit der Notwendigkeit von einer externen Notstromversorgung bis hin zu Evakuierungen nicht ausgeschlossen und wurden wiederholt beschrieben [3, 26].

In Umfragen konnte weiterhin gezeigt werden, dass auch nach einem erlebten Stromausfall die private Vorratshaltung mangelhaft war. Dies betrifft auch die Informiertheit der Bevölkerung und deren Risikobewusstsein [10, 25]. Somit bleibt festzustellen, dass ein Stromausfall mit einer Vielzahl von Herausforderungen einhergeht und gleich zu Beginn medizinische Schwerpunkte identifiziert werden müssen. Hier muss dann in Abhängigkeit von den regionalen Gegebenheiten entsprechend reagiert werden. Im Rahmen der Vorplanungen spielt einerseits die Hilfe zur Selbsthilfe eine Rolle, andererseits kann es sinnvoll sein, entsprechende Pläne zu erstellen, die Informationen zu speziellen Pflegeheimen oder besonders hilfsbedürftigen Patienten beinhalten. Hier konnte gezeigt werden, dass von zu Pflegenden 79 % bereit wären, ihre Daten bei einer Behörde zu hinterlegen [30]. Die Möglichkeit zur kurzfristigen Schaffung temporärer Pflegeeinrichtungen kann sinnvoll sein.

Entsprechende Projekte, die die Vernetzung von Behörden, Pflegeinfrastrukturen, Angehörigen und zivilgesellschaftlichen Akteuren im Hinblick auf derartige Schadensereignisse erproben, sind bereits etabliert („Kontexte von Pflege und Hilfsbedürftigen Stärken“ [7]); vergleichbare Ansätze sollten auch im großstädtischen Raum erfolgen.

### Infobox 1 Notstromversorgung im Krankenhaus. (Modifiziert nach [12])

- Not-OP, inklusive Lüftung
- Intensivstationen (Beatmungsgeräte)
- Kühlung von Blutkonserven und Organen
- Heizungs- und Wasserpumpen
- Notbeleuchtung
- Aufzüge zum Patiententransport
- Belüftung in sensiblen Bereichen
- Sterilisation

### Infobox 2 Vulnerable Gruppen bei einem Stromausfall. (Modifiziert nach [18])

- Alte Menschen
- Behinderte Menschen
- Psychisch Kranke/chronisch Kranke
- Drogenabhängige
- Mütter mit Neugeborenen
- Obdachlose
- Geflüchtete
- Fremdsprachige Touristen

### Infobox 3 Medizinische Geräte, die stromabhängig bzw. akkubetrieben sind

- Heimbeatmungsgerät
- Kunstherz
- Inhalationsgerät
- Heimdialysegerät
- Ernährungspumpe
- (Blutzuckermessgerät)
- (Insulinpumpe)

## Fazit für die Praxis

- Ein großflächiger Stromausfall erfordert eine professionelle, interdisziplinäre Zusammenarbeit aller beteiligten Akteure. Neben der Einberufung eines operativ-taktischen Stabes müssen in diesem v. a. auch frühzeitig durch fachlich adäquat besetzte Funktionen medizinische Schwerpunkte identifiziert werden. Umfangreiche Krisenkommunikation mit Anleitungen zur Selbsthilfefähigkeit der Bevölkerung ist notwendig.
- Die schnelle Verfügbarkeit von zusätzlichen materiellen und personellen Ressourcen der Notfallrettung ist entscheidend für die Systemstabilität bei derartigen Schadenslagen.
- Vulnerable Gruppen müssen im Schadensgebiet identifiziert werden. Gerade netzstromabhängige pflegebedürftige Menschen, die in der Häuslichkeit oder in Wohngemeinschaften versorgt werden, sind nur unzureichend registriert.
- Ein besonderes Augenmerk muss auf den Funktionserhalt von Krankenhäusern als Bestandteil der kritischen Infrastruktur gelegt werden.
